# Bergamottin and 5-Geranyloxy-7-methoxycoumarin Cooperate in the Cytotoxic Effect of *Citrus bergamia* (Bergamot) Essential Oil in Human Neuroblastoma SH-SY5Y Cell Line

**DOI:** 10.3390/toxins13040275

**Published:** 2021-04-10

**Authors:** Alessandro Maugeri, Giovanni Enrico Lombardo, Laura Musumeci, Caterina Russo, Sebastiano Gangemi, Gioacchino Calapai, Santa Cirmi, Michele Navarra

**Affiliations:** 1 Department of Chemical, Biological, Pharmaceutical and Environmental Sciences, University of Messina, 98168 Messina, Italy; amaugeri@unime.it (A.M.); gelombardo@unime.it (G.E.L.); laura.musumeci@unime.it (L.M.); carusso@unime.it (C.R.); mnavarra@unime.it (M.N.); 2 Fondazione “Prof. Antonio Imbesi”, 98168 Messina, Italy; 3 Department of Clinical and Experimental Medicine, University of Messina, 98168 Messina, Italy; sebastiano.gangemi@unime.it; 4 Department of Biomedical and Dental Sciences and Morphofunctional Imaging, University of Messina, 98168 Messina, Italy; gioacchino.calapai@unime.it; 5 Department of Pharmacy-Drug Sciences, University of Bari “Aldo Moro”, 70125 Bari, Italy

**Keywords:** *Citrus bergamia*, bergamot, cancer, coumarin, psoralen, pharmacological combination, bergamottin, 5-geranyloxy-7-methoxycoumarin

## Abstract

The plant kingdom has always been a treasure trove for valuable bioactive compounds, and *Citrus* fruits stand out among the others. Bergamottin (BRG) and 5-geranyloxy-7-methoxycoumarin (5-G-7-MOC) are two coumarins found in different *Citrus* species with well-acknowledged pharmacological properties. Previously, they have been claimed to be relevant in the anti-proliferative effects exerted by bergamot essential oil (BEO) in the SH-SY5Y human neuroblastoma cells. This study was designed to verify this assumption and to assess the mechanisms underlying the anti-proliferative effect of both compounds. Our results demonstrate that BRG and 5-G-7-MOC are able to reduce the proliferation of SH-SY5Y cells, inducing apoptosis and increasing cell population in sub-G0/G1 phase. Moreover, we demonstrated the pro-oxidant activity of the two coumarins that increased reactive oxygen species and impaired mitochondrial membrane potential. From a molecular point of view, BRG and 5-G-7-MOC were able to modulate apoptosis related factors at both protein and gene levels. Lastly, we evaluated the synergistic effect of their combination, finding that the highest synergy was observed at a concentration ratio similar to that occurring in the BEO, supporting our initial hypothesis. Taken together, our results deepen the knowledge regarding the effect of BRG and 5-G-7-MOC in SH-SY5Y cells, emphasizing the relevance of their cooperation in achieving this effect.

## 1. Introduction

Our planet is a treasure trove of uncountable substances which are constantly under unceasing investigation of researchers. These compounds can be beneficial or harmful to human health, yet, if many of them were primarily considered just toxic for humans, they are now proved to be therapeutically relevant. It was in the 17th century, when Paracelsus in his “Opera omnia” stated “Dosis sola facit, ut venenum non sit” (“Only dose makes the poison”), that one of the pillars of modern toxicology was established. Famous evidence of this postulate are natural drugs employed in chemotherapy (i.e., *Vinca* alkaloids, podophyllotoxins, camptothecins, taxanes, etc.) which were obtained from “poisonous” plants, nonetheless representing now the first line treatments for many solid or hematologic tumors [1]. Despite this resounding example, the toxicity of several classes of compounds extracted from various plants still overcome their potential therapeutical application in pathologies like cancer. In this regard, essential oils represent a noticeable source of active principles that are worthy of extensive studies. Among these, monoterpenes are the main components of plant essential oils and hence employed in a multitude of fields, from food to cosmetic and perfume industries, for their olfactory properties. These compounds are claimed to be hepatotoxic due to their fast absorption by passive transport and their metabolization at liver level into more toxic products [2], nevertheless many studies have proved that monoterpenes possess interesting biological effects such as antiviral, antifungal as well as antitumoral [3]. Coumarins are other secondary plant metabolites that are mainly found in essential oils, even though in much lesser quantities respect to monoterpenes. These compounds are present in food and used mostly as additives in cosmetic and perfume industry. The exposure to coumarins is acknowledged to induce moderate hepatotoxic and carcinogenic effects [4]. Nonetheless, they have been claimed also to possess anticancer properties, as already reviewed [5]. Furocoumarins, compounds that differ from those abovementioned by a fused furan ring to the 1-benzopyran-2-one scaffold, and employed for the same aims as those above, exhibit phototoxicity especially when interacting with ultraviolet A (UVA) radiation, thus triggering cytotoxic and mutagenic effects. Notably, this phototoxicity is currently exploited in the so-called PUVA therapy, where “P” stands for psoralen, a linear furocoumarin [6]. Given the potentiality of essential oils, we previously evaluated the mechanisms underlying the antiproliferative effect of *Citrus bergamia* (bergamot) essential oil (BEO), suggesting that bergamottin (BRG) and 5-geranyloxy-7-methoxycoumarin (5-G-7-MOC) played a crucial role in BEO activity towards this cell line [7]. Following this, the aim of this work was to deepen the knowledge concerning the activity of these two compounds and their role in the effect of the whole BEO.

## 2. Results

### 2.1. Bergamottin (BRG) and 5-geranyloxy-7-methoxycoumarin (5-G-7-MOC) Induce Cytotoxic Effects in SH-SY5Y Cells

**Figure 1:**
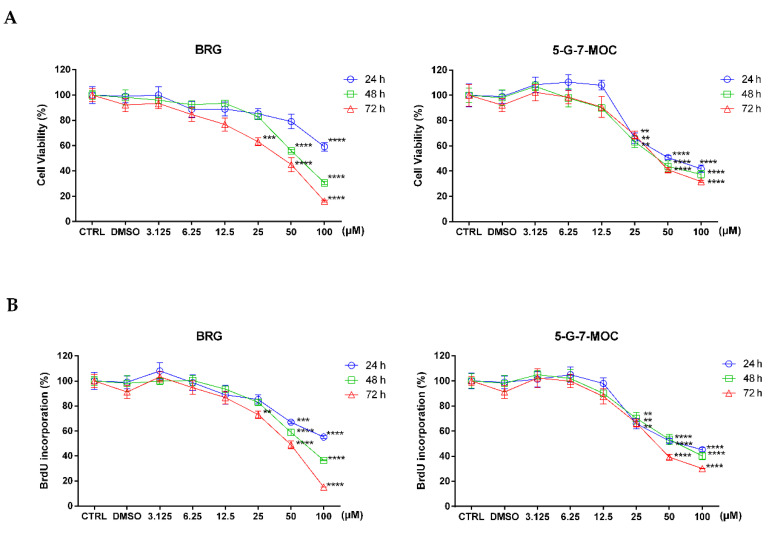
Effects of BRG and 5-G-7-MOC (3.125 to 100 µM) on SH-SY5Y neuroblastoma cell proliferation for 24–72 h. Viability rate was assessed by MTT (**A**) and BrdU incorporation (**B**) tests. Results of both assays are expressed as percentages ± standard error of the means (SEM) of the absorbance values detected in the control (CTRL) cells. Each concentration was tested eight-fold and three independent experiments were carried out (*n* = 24). ** *p* < 0.01, *** *p* < 0.001 and **** *p* < 0.0001 vs. CTRL.

The effects on cell proliferation of BRG and 5-G-7-MOC were assessed by the 4,5-dimethylthiazol-2-yl)-2,5-diphenyltetrazolium bromide (MTT; Figure 1A) test, index of mitochondrial functionality, and 5-bromo-2′-deoxyuridine (BrdU; Figure 1B) assay, index of DNA replication. As shown in Figure 1, both compounds were able to hamper viability of SH-SY5Y cell line significantly already at 25 µM, despite to different extent. This outcome was clear both in the MTT and BrdU incorporation tests. The IC_50_s extrapolated from the curves were 36.8 ± 3.8 µM for BRG and 46.9 ± 5.47 µM for 5-G-7-MOC at 72 h of treatment. In line of these results, 25 and 50 µM were chosen as concentrations for further studies. The percentage of dimethyl sulfoxide (DMSO; 0.2%) present in the highest concentration (100 µM) served as vehicle control to confirm that no effect was caused by the solvent.

### 2.2. BRG and 5-G-7-MOC Promote Apoptosis in SH-SY5Y Cells

**Figure 2:**
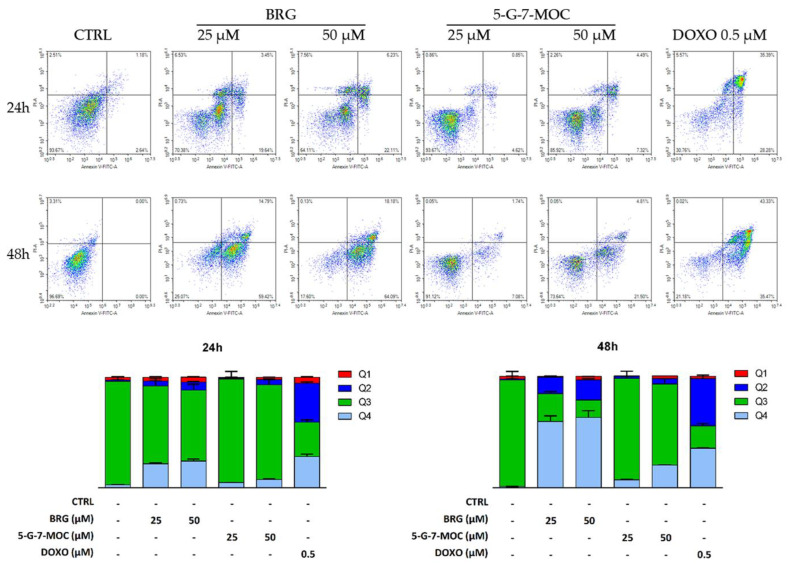
Fluorescence-activated cell sorting (FACS) analysis of apoptosis in SH-SY5Y cells exposed to BRG and 5-G-7-MOC. The detection of apoptosis was performed through the Annexin V-FITC/PI test. Representative Annexin V vs PI dot plots of the SH-SY5Y cells treated with 25 and 50 μM of both compounds for the indicated periods are displayed. Q3 contains the viable cells (Annexin V −/PI −), Q4 contains the cells in early apoptosis (Annexin V +/PI −), Q2 contains the cells in late apoptosis (Annexin V +/PI +), while Q1 contains the necrotic ones (Annexin V −/PI +). Histograms depict the percentages of cells present in the corresponding quadrants ± SEM of three experiments separately performed in triplicate (*n* = 9).

In order to ascertain the type of cell death induced by BRG and 5-G-7-MOC in SH-SY5Y cells, the Annexin V-fluorescein isothiocyanate (FITC)/propidium iodide (PI) cytofluorimetric assay was performed. The treatment with BRG at 25 and 50 μM increased the percentage of cells undergoing apoptosis, both early and late, up to 23.1% and 29% (24 h) and up to 69.6% and 84.4% (48 h), respectively (Figure 2). In parallel, 5-G-7-MOC treatment induced a lesser increase of apoptotic cells, compared to the other coumarin: 6.6% and 14.2% (24 h), while 11.5% and 32.2% (48 h) were recorded for 25 and 50 μM, respectively (Figure 2).

### 2.3. BRG and 5-G-7-MOC Force SH-SY5Y Cells out of the Cell Cycle

**Figure 3:**
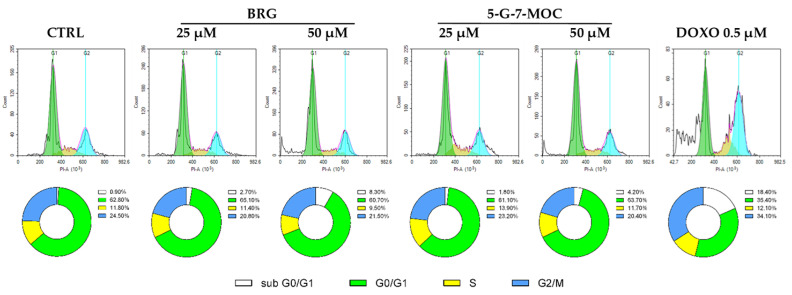
Impact of BRG and 5-G-7-MOC on cell cycle progression of SH-SY5Y cells. The outcomes of the treatment for 48h with BRG and 5-G-7-MOC at 25 and 50 µM on cell cycle of SH-SY5Y cells were appreciated by the propidium iodide assay through flow cytometry. The plots are representative of three different experimental sessions performed in triplicate (*n* = 9). Percentages of cells present in each phase of the cell cycle are reported in the donut charts (sub G0/G1: white; G0/G1: green; S: yellow; G2/M: blue).

By the PI assay, the effect of BRG and 5-G-7-MOC on the cell cycle progression of SH-SY5Y cells was evaluated. The two compounds did not alter the ratio among G0/G1, S and G2/M phases, regardless of concentrations or times tested. Nonetheless, the treatment for 48 h of SH-SY5Y cells with BRG and 5-G-7-MOC increased the percentage of cells in sub-G0/G1, phase in which apoptotic cells gather and hence exit the cell cycle, up to 2.7% and 8.3% for BRG 25 µM and 50 µM, respectively, and up to 1.8% and 4.2% for 5-G-7-MOC 25 µM and 50 µM, respectively (Figure 3).

### 2.4. BRG and 5-G-7-MOC Increase Reactive Oxygen Species (ROS) Production and Impair Mitochondrial Membrane Potential (∆Ψm)

**Figure 4:**
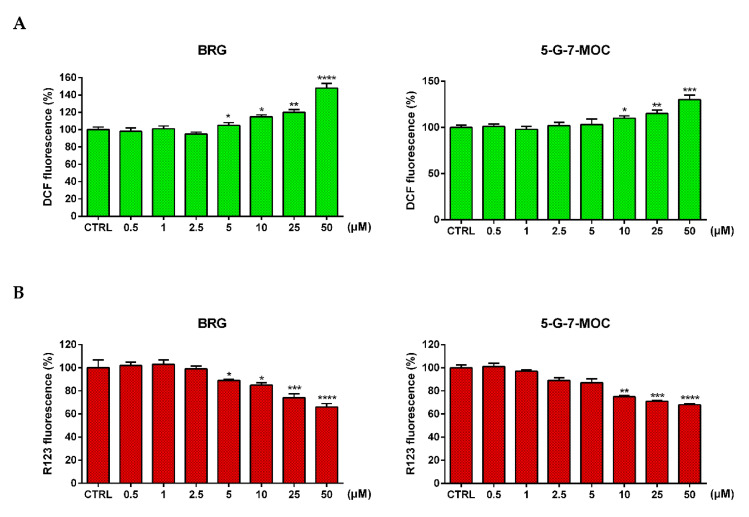
Generation of ROS and fall of ΔΨm in SH-SY5Y after treatment with BRG and 5-G-7-MOC. (**A**) ROS levels were assessed through the fluorescent probe DCFH-DA. (**B**) Variations of ΔΨm were evaluated through the cationic fluorochrome R123. Results of both assays are expressed as percentages ± SEM of the fluorescence values detected in the control cells of three different experiments for eight replicates (*n* = 24). * *p* < 0.05, ** *p* < 0.01, *** *p* < 0.001 and **** *p* < 0.0001 vs. CTRL.

The production of ROS in SH-SY5Y cells exposed to BRG and 5-G-7-MOC at 25 and 50 µM for 24 h was evaluated employing the probe 2′,7′-dichlorodihydrofluorescein diacetate (DCFH-DA), which becomes fluorescent in the presence of intracellular radicals. As shown in Figure 4A, both compounds were able to significantly increase ROS after 24 h of treatment in our cellular model already at 5 and 10 µM for BRG and 5-G-7-MOC, respectively (Figure 4A). To assess the effect of the two coumarins on the ∆Ψm, we exploited the rhodamine 123 (R123) as probe. In accordance to the previous assay, BRG and 5-G-7-MOC decreased significantly the ∆Ψm of treated SH-SY5Y from 5 and 10 µM for BRG and 5-G-7-MOC, respectively (Figure 4B).

### 2.5. BRG and 5-G-7-MOC Modulate Apoptosis-Related Factors in SH-SY5Y Cells at Both Gene and Protein Level

**Figure 5:**
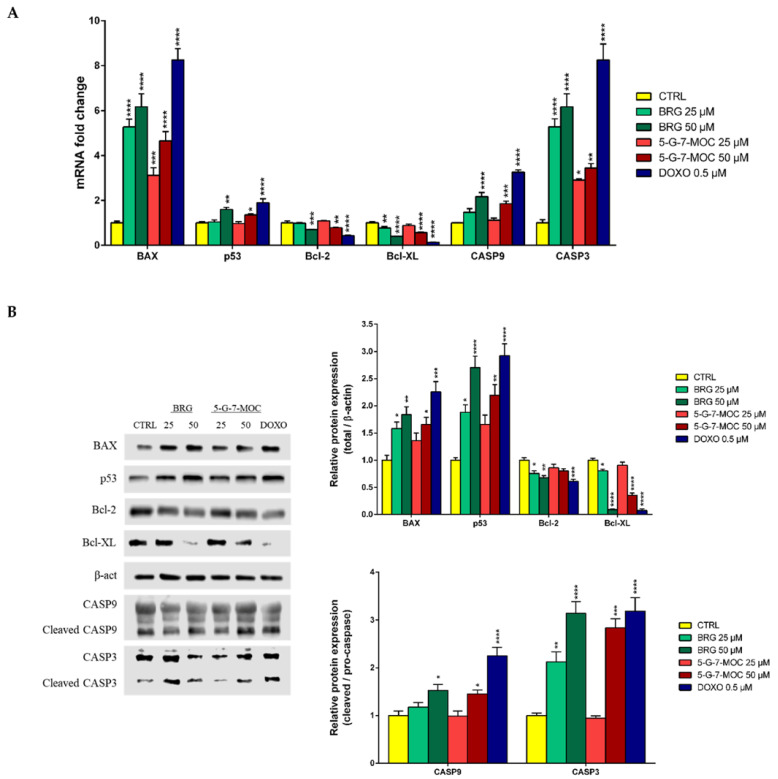
Modulation of apoptosis-related gene and protein levels in SH-SY5Y cells treated with BRG and 5-G-7-MOC. Cells exposed to BRG and 5-G-7-MOC at 25 and 50 µM for 24 h were processed for mRNA and protein expression studies. (**A**) Relative quantities of mRNA, obtained through real-time PCR made in triplicate, were calculated by the 2^–∆∆Ct^ method, with β-actin as housekeeping gene. (**B**) Immunoblots of proteins, obtained from Western blotting studies, are shown along with their densitometric analyses, on the right. The expression of BAX, p53, Bcl-2 and Bcl-XL was normalized against β-actin (β-act), while that of both caspases is expressed as ratio of the cleaved form with respect to the relative zymogen. Results are expressed as fold change respect to untreated cells and expressed as mean ± SEM of three different sets of experiments performed in triplicate (*n* = 9). * *p* < 0.05, ** *p* < 0.01, *** *p* < 0.001 and **** *p* < 0.0001 vs. CTRL.

To study the pathways involved in the anti-cancer effect of the two coumarins in SH-SY5Y cells, we evaluated both gene and protein levels of the most pivotal factors linked to apoptosis. The treatment with BRG and 5-G-7-MOC at 25 and 50 µM for 24 h brought an increase of gene expression of the pro-apoptotic B-cell lymphoma (Bcl)-2-associated X protein (BAX), p53 and both caspases 9 (CASP9) and 3 (CASP3), as well as a decrease of Bcl-2 and Bcl-XL (Figure 5 A). In particular, the aforementioned outcome was significant for BRG and 5-G-7-MOC at the highest concentration tested (50 µM), despite to different strength. The lowest concentration (25 µM), instead, reached a significant result only for BAX and CASP3 genes. These results reflected also at protein level, as assessed by Western blot (Figure 5B). The treatment of SH-SY5Y with both concentrations of BRG showed a significant increase of both BAX and p53 and a decrease of Bcl-2 and Bcl-XL protein levels. Moreover, the cleavage of pro-caspases 9 and 3 was significantly induced by BRG 50 µM, while only for CASP3, as regards to BRG 25 µM. In parallel, only the highest concentration of 5-G-7-MOC (50 µM) was able to provide a significant effect on the protein levels of all the factors here studied, except for Bcl-2 which was slightly decreased by 5-G-7-MOC treatment, though not significantly.

### 2.6. BRG and 5-G-7-MOC Synergistically Cooperate to Induce Cytotoxic Effects in SH-SY5Y Cells

**Figure 6:**
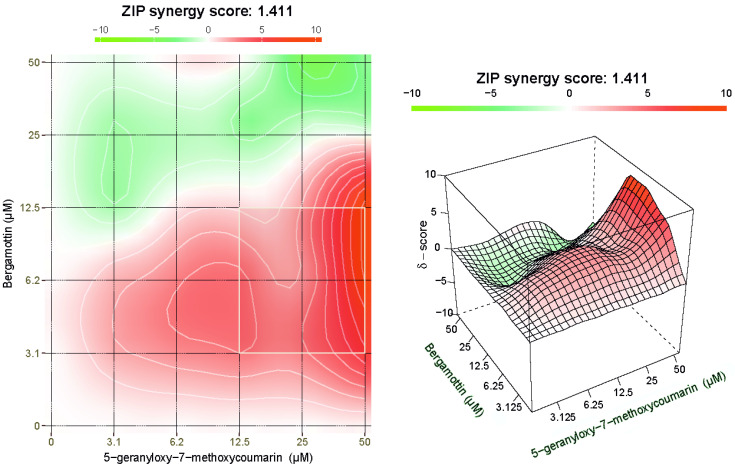
Synergistic effect of BRG and 5-G-7-MOC treatment in SH-SY5Y cells. Cell viability was assessed by MTT assay and the effect of the drug combination was calculated and visualized using SynergyFinder 2.0 software through the ZIP reference model. The synergy score is expressed as the mean of all δ scores for the dose-response analysis, and the maps shown are representative of three different experimental sessions. The red, white and green regions of the graph indicate the synergy, additivity and antagonism, respectively. The white square represents the highest synergistic area.

The synergism between the two coumarins was evaluated testing their combination at different ratios and assaying the inhibition of cell viability through MTT test, whose results have been processed by SynergyFinder 2.0 software (Figure 6). The combination of BRG and 5-G-7-MOC proved to be overall synergistic with a zero interaction potency (ZIP) score of 1.411, despite only for certain ratios. In particular, when these were between 1:4 to 1:16, we observed the highest δ scores (δ = 4.52), and hence synergism (red area). Conversely, the treatment of SH-SY5Y cells with equimolar concentrations of BRG and 5-G-7-MOC or at a ratio of 2:1 led to a strong antagonistic effect (green area).

## 3. Discussion

*Citrus* fruits (CF) have represented valuable tools to improve one self’s health for centuries, thanks to the extraordinary variety of compounds present in them which make these fruits precious allies to fight or prevent different illnesses. Each part of these fruits provides different active principles: the flavedo (i.e., the outer peel), from which the essential oil is obtained, is plenty of monoterpenes and coumarins; the albedo, (i.e., the white spongy portion of the fruit) is full of polyphenols as well as the juice, which is also rich of other micronutrients [8]. Therefore, CF and their juices have been widely studied for their protective activities against oxidative stress and inflammation [9,10,11], cardiovascular [12] and neurodegenerative diseases [13,14], microbial infections [15], and some types of cancer [16,17,18]. Essential oils of CF were also the focus of several studies aimed at evaluating their properties [19]. Among the great variety of CF present worldwide, *Citrus bergamia* (bergamot) Risso & Poiteau, a member of the family of Rutaceae, has drawn the attention of the scientific community for its properties. From squeezing its fruit, we obtain the juice (BJ), used mostly in the food industry. BJ has been studied, among others, for its antimicrobial potential [20] and anticancer activity in in vitro [21,22] and in vivo [23,24] experimental models, suggesting its high flavonoid content as the responsible for this effect [25]. Consequently, further studies were aimed at assessing the flavonoid-rich extract of BJ (BJe). It has been claimed that BJe possesses antioxidant activity [26,27] and is able to hinder inflammation, both in vitro [28,29] and in vivo [30,31,32]. BJe interacted also with the AMPK/SIRT1 axis [33], indicating its potential role as a remedy for inflammation-based illnesses [34]. BEO, the most important product obtained by bergamot fruits and used mostly in perfume industry, is a phytocomplex composed by a volatile fraction, (almost the 95% of the total) that consists of monoterpenes like limonene, linalool, linalyl acetate, α-pinene, β-pinene, and γ-terpinene, and a non-volatile one, that consists mostly of coumarins and psoralens, like bergapten, bergamottin and citropten [35]. It was studied for its application in aromatherapy [36], as well as for its antimicrobial [15], cardioprotective [37], and anticancer properties [7,38].

In our study, we started from the fact that BEO was able to induce anti-proliferative effect in human neuroblastoma SH-SY5Y cells as whole [7]. Interestingly, in the same study, we also evaluated the effect of fractions of BEO deprived of furocoumarins or terpenes, which proved to be almost as effective as the whole BEO. Focusing on the quali-quantitative composition of those fractions, we saw that linalool and linalyl acetate, among volatile compounds, as well as BRG and 5-G-7-MOC, among coumarins, were in common among the three studied fractions. Although Russo and co-workers [39] claimed that those monoterpenes were inactive in SH-SY5Y cell line regardless the high concentrations employed for their study, many other studies reported the effects of linalool and linalyl acetate in different experimental models [3,40,41,42,43]. In this line, we assessed those monoterpenes at the concentration present in the BEO at the highest concentration tested previously [7]. We proved that, after just 24 h, they showed a strong anti-proliferative activity alone and in combination, without noticing any noteworthy difference (data not shown). From this, we deduced that these monoterpenes are definitely the major players in the anti-proliferative activity of BEO. When we tested BRG and 5-G-7-MOC at the concentration present in BEO (0.03%) for 24 h, we saw no significant anti-proliferative effect either alone or in combination together. Interestingly, when we put together linalool, limonene or linalyl acetate with the mixture of coumarins, the cytotoxic effect increased with respect to the combination of monoterpenes alone. This confirmed our hypothesis on their interesting role in the BEO effect [7] and led us investigate further on their activity in neuroblastoma cell line. The anti-cancer activity, as well as the underlying mechanisms, of BRG was previously evaluated in different in vitro and in vivo models [44], whereas that of 5-G-7-MOC was reported only in colon cancer cells [45]. In SH-SY5Y cells, we are the first to report that both compounds are able to induce anti-proliferative activity, though with different efficacy, with an IC_50_ of 36.8 ± 3.8 µM for BRG and 46.9 ± 5.47 µM for 5-G-7-MOC, respectively. Subsequently, to assess which type of cell death these coumarins induced in neuroblastoma cells, we firstly investigated whether our compounds interfere with the progression of SH-SY5Y cells during cell cycle through cytofluorimetric analyses. Cell cycle is characterized by checkpoints strictly complied by normal cells; in case genomic aberrations or defects occur, cells undergo cell cycle arrest [46]. In our study, neither BRG nor 5-G-7-MOC were able to alter the ratio among the G0/G1, S and G2/M phases with respect to controls for any of the tested concentrations. On the other hand, we witnessed an increase of the cell population in the sub-G0/G1 phase, typical of hypodiploid cells, after the treatment with BRG and, though to a slighter extent, 5-G-7-MOC, thus suggesting that these coumarins might push cells to exit cell cycle and go into apoptosis. For this reason, we verified our hypothesis through Annexin-V/PI staining, a specific assay aimed at evaluating whether the apoptotic process started and at which stage (i.e., early or late) cells are. Apoptosis is a process finely regulated in healthy cells; hence, its dysregulation represents a well-known tumoral marker [47]. In this experimental model, BRG was able to induce apoptosis in SH-SY5Y cells at both 25 and 50 µM already at 24 h of treatment, whereas 5-G-7-MOC only at 48 h.

Oxidative balance in normal cells is precisely balanced, as well in tumors. However, in cancer cells, ROS levels are higher as a mechanism of keeping the process of DNA impairment continuing. Nevertheless, pushing ROS levels even further in those cells is known to start a vicious cycle. This is because overcoming anti-oxidant defense will lead to an extensive DNA damage, deposition of Bax protein on mitochondrial membrane, a decrease of its potential, and hence, additional ROS production [48]. Here, BRG and 5-G-7-MOC induced ROS overproduction in our in vitro model, as well as, the impairment of ∆Ψm.

From a molecular point of view, one of the triggers of apoptosis can be an irreparable DNA damage that subsequently activates downstream proteins aimed at regulating this event. Among these, p53 represents one of the most relevant promotors of the apoptotic machinery which, after genomic impairment, triggers downstream proteins like BAX and Bad, well-known pro-apoptotic factors, and hinders those of the Bcl family (i.e., Bcl-2 and Bcl-XL), well-known anti-apoptotic ones. These proteins work together to activate or inhibit the caspase cascade, which in turn leads to apoptosis [49]. In our study, we witnessed a decrease of Bcl-2 and Bcl-XL, whereas BAX and p53 increased, as a clear sign of cells going towards apoptosis. This was coupled to the cleavage, and hence activation, of both caspases 9 and 3, indicating that BRG and 5-G-7-MOC follow the intrinsic apoptotic pathway. The effect observed at protein level by results of Western blotting analyses were confirmed at gene level by real-time PCR studies.

After proving the anti-proliferative effect of both BRG and 5-G-7-MOC alone in neuroblastoma cell line, we wondered how these two compounds might affect the activity of the whole BEO, as we highlighted before, given the fact that they are at concentrations in the essential oil far below from the IC_50_s extrapolated in this study. Therefore, we evaluated whether their combination could be synergistic or antagonistic through the employment of a computational methodology. Several approaches have been established so far to assess pharmacological interdependence among drugs; the Loewe additivity and Bliss independence drug interaction models are the most relevant and accepted, whereas the zero interaction potency (ZIP) is a newer protocol derived from the previous ones [50]. The synergy scores obtained shed light on the fact that the most synergistic combinations between BRG and 5-G-7-MOC are when the latter’s concentration is lower than the former’s one, more specifically with ratios ranging from 1:4 to 1:16. Remarkably, in the BEO, as well as in the fractions studied before [7], the ratio between them is about 1:14, value that falls perfectly within the range of the most synergistic area of the maps obtained by drug combination software. Therefore, this explains why, despite the low concentrations of the two coumarins in BEO, they play together a relevant role in its activity, supporting the monoterpene counterpart. This outcome assumes a relevant connotation since it is well-known that synergy among compounds present in natural products is crucial given their complex nature [51], and hence their simultaneous multitarget capacity may lead to greater effect than that achievable by single molecules [9,17,33]. Therefore, when using phytocomplexes, from a toxicological point of view, not only the relative quantity of a determined compound should be taken into account, but also the potential synergy with the others. On the other hand, from a potential therapeutic approach, the synergism in natural products is an exceptional tool due to the fact that stronger effects can be obtained by employing lower concentrations [52,53].

## 4. Conclusions

This study deepens the knowledge regarding the role of BRG and 5-G-7-MOC in the anti-proliferative effect of BEO in SH-SY5Y cell line, investigating their cooperation to achieve this outcome.

## 5. Materials and Methods

### 5.1. Cell Culture and Treatments

The human neuroblastoma cell line SH-SY5Y was originally obtained from ATCC (Rockville, MD, USA). The cells were cultured were cultured in RPMI supplemented with 10% (*v*/*v*) heat-inactivated fetal bovine serum, L-glutamine (2 mM), sodium pyruvate (1 mM), penicillin (100 IU/mL), and streptomycin (100 µg/mL). Each cell culture reagent was from Gibco (Life Technologies, Monza, Italy). BRG and 5-G-7-MOC were from Extrasynthese (Genay, France). The stock solutions (50 mM) of BRG and 5-G-7-MOC were prepared in DMSO, which were employed to obtain the working concentrations, upon further dilution in culture medium.

### 5.2. Cell Viability and Proliferation Assays

For the evaluation of the anti-proliferative activity, we employed the MTT test, as previously described [54,55]. Briefly, SH-SY5Y cells were seeded in 96-well plates at a density of 5 × 10^3^ cells/well. The day after, cells were treated with media containing the desired concentrations of BRG and 5-G-7-MOC; in untreated cultures, we only changed the medium. We then incubated the plates for further 24, 48, and 72 h, centrifuged them and replaced media with fresh one without phenol red and containing 0.5 mg/mL of MTT (Sigma-Aldrich, Milan, Italy). Plates were left to incubate for 4 h until the crystals of formazan formed in the wells. The supernatants were removed after centrifugation and crystals solubilized in 100 µL of 0.1 N HCl/isopropanol lysis solution. The absorbance was recorded by a microplate spectrophotometer at 570 nm with reference at 630 nm (iMark™ microplate reader, Bio-Rad Laboratories, Milan, Italy). Results were extrapolated as percentage of cell viability respect to untreated cells. For the BrdU incorporation, as marker of cell proliferation, we employed the BrdU Cell Proliferation Assay Kit (Merck Millipore, Darmstadt, Germany) and followed manufacturer’s guidelines, as reported [56]. Similar to MTT assay, after treatments, cells were exposed to BrdU for 2 h, then fixed and washed, prior adding the anti-BrdU peroxidase conjugated-antibody. Finally, we added the provided substrate and stopped the reaction in order to detect results with a microplate spectrophotometer at 450 nm (iMark™ microplate reader, Bio-Rad Laboratories). Similar to MTT, results were extrapolated as percentage of cell proliferation respect to untreated cells.

### 5.3. Cytofluorimetric Analyses

Fluorescence-activated cell sorting (FACS) was exploited to evaluate the role of the tested compounds in inducing apoptosis or interfering with the progression of cell cycle [57,58].

#### 5.3.1. Apoptosis Evaluation

Annexin V-FITC/PI staining is a common procedure to assess whether apoptotic machinery is involved or not, given the high affinity of Annexin V towards phosphatidylserine exposed in early stages of this process and of PI towards the DNA. Briefly, 10 × 10^3^ SH-SY5Y cells were seeded in 6-well plates and let adhere for 24 h. The next day, cells were treated with 25 and 50 µM of BRG and 5-G-7-MOC for further 24 and 48 h. Afterwards, cells were collected by trypsinization, washed with PBS and resuspended in 1× binding buffer, following kit guidelines (eBioscience, Thermo Fisher Scientific, Monza, Italy). Then, each sample was resuspended in 200 µL 1× binding buffer, with both 5 μL of Annexin-V-FITC and 10 μL of PI, gently vortexed and incubated at room temperature in darkness for 15 min. Samples were run on a Novocyte 2000 cytofluorimeter (ACEA Bioscences Inc., San Diego, CA, USA). Doxorubicin 0.5 µM was employed as positive control.

#### 5.3.2. Cell Cycle Evaluation

PI stoichiometrically binds to DNA allowing its precise quantification and discrimination among cell phases. Similar to apoptosis evaluation, SH-SY5Y cells were seeded at a density of 10 × 10^3^ cells in 6-well plate, treated accordingly for 48 h with 25 and 50 µM of BRG and 5-G-7-MOC, and harvested by trypsinization. Then cells were washed with PBS and resuspended in 70% ice-cold ethanol while gently vortexed. After at least 4 h at 4 °C, cells were washed twice and RNA was digested by RNase (10 mg/mL in PBS) at 37 °C for 1h. Finally, 10 µL of PI (1 mg/mL; Sigma-Aldrich, Milan, Italy) were added to samples and immediately acquired by flow cytometry. Doxorubicin 0.5 µM was employed as positive control.

### 5.4. Determination of ROS and ∆ψm

ROS and Δψm were measured fluorometrically as oxidative stress markers [14]. SH-SY5Y cells were seeded in 96-well plates at a density of 5 × 10^4^ cells/well, allow to adhere and, the following day, treated with increasing concentrations of BRG and 5-G-7-MOC (1–50 µM) for 24h. The quantification of ROS was obtained employing the probe DCFH-DA (25 µM; Sigma-Aldrich), while the evaluation of Δψm by R123 (10 µM; Sigma Aldrich) as reported [12]. The fluorescence was acquired by a microplate reader (POLARstar Omega, BMG Labtech, Ortenberg, Germany; 485 nm Ex. and 535 nm Em. for DCFH-DA; 488 nm Ex. and 525 nm Em. for R123).

### 5.5. Real-Time PCR

SH-SY5Y cells were seeded in 100 mm Petri dishes at a density of 1 × 10^6^ cells/dish and, after 24 h, treated with BRG and 5-G-7-MOC (25 and 50 µM) for 24 h. Doxorubicin 0.5 µM was used as positive control. The day after, total RNA was extracted using TRIzol reagent, according to the manufacturer’s protocol. Then, equal amounts of extracted RNA (2 μg) were reverse transcribed using a High-Capacity cDNA Archive Kit (Applied Biosystems, Thermo Fisher, Foster City, CA, USA). The mRNA levels of Bax, Bcl-2, Bcl-Xl, p53 and CASP3/9 were assessed by Real-Time PCR (qPCR). The reactions were performed in a 96-well plate in 20 μL containing 1× SYBR^®^ Premix Dimer Eraser™ (TaKaRa Bio Inc., Tokyo, Japan), 0.1 μM specific primers and 25 ng RNA converted into cDNA. The qPCR was carried out in a 7300 qPCR System (Applied Biosystems, Thermo Fisher) with the next profile: one cycle at 95 °C for 10 min, followed by 40 cycles at 95 °C for 15 s, and 60 °C for 1 min. A standard dissociation stage was added to assess the primer specificity. β-Actin was used as the housekeeping control. The primer sequences are listed in the Table 1. The collected data were evaluated using the 2^−ΔΔCT^ relative quantification method [59].

### 5.6. Western Blot

SH-SY5Y cells were seeded in 100 mm Petri dishes at a density of 1 × 10^6^ cells/dish, allow to adhere overnight and then cells were treated with BRG and 5-G-7-MOC (25 and 50 µM) for 24 h. Doxorubicin 0.5 µM was used as positive control. Total protein content was evaluated as previously described [60,61], using a Bio-Rad Protein Assay (Bio-Rad Laboratories) and bovine serum albumin as standard. Proteins (30 μg/lane) were separated by 10% sodium dodecyl sulphate-polyacrylamide gel electrophoresis (SDS-PAGE), and then transferred on polyvinylidene difluoride (PVDF; Merck Millipore). Non-specific binding sites were blocked with 5% non-fat milk for 1 h and incubate overnight at 4°C with the following primary antibodies: mouse anti-Bcl-2 (Santa Cruz Biotechnology, Segrate, Milan, Italy); rabbit anti-Bax (GeneTex, Irvine, CA, USA) and rabbit anti-Bcl-XL, anti-p53, anti-CASP3 and anti-CASP9 (Cell Signaling Technology, Danvers, MA, USA); rabbit anti-β-actin (Sigma-Aldrich). Membranes were washed thrice and incubated with horseradish peroxidise-conjugated goat anti-mouse or anti-rabbit IgG secondary antibodies (Santa Cruz Biotechnology) at room temperature for 1 h. Chemiluminescence of protein bands was obtained employing Luminata Forte Western HRP Substrate (Merck Millipore) and quantified with C-Digit Blot Scanner (Li-COR Bioscience, Lincoln, NE, USA). The protein expression was quantified using Image Studio software (Li-COR Bioscience). The β-actin was used as housekeeping protein.

### 5.7. Drug Combination Treatments and Analysis of Synergistic Effect

The effects of the combination between BRG and 5-G-7-MOC was assessed through the checkboard method by which SH-SY5Y cells, seeded in the same manner as for cell proliferation assays, were treated with increasing concentrations (1–50 µM) of the abovementioned compounds combined together at different ratios. Cell viability was assessed by MTT assay and results processed to determine their interactions.

Synergy scoring was determined using the SynergyFinder 2.0 software that exploits the ZIP calculation method, expressing the synergism as δ score. Positive δ values correspond to synergism, whereas negative ones to antagonism [50,62].

### 5.8. Statistical Analysis

One-way and two-way analyses of variance (ANOVA) were employed to analyze data, depending on the assay, which are expressed as mean ± standard error of the means (SEM). Multiple comparisons of the means of the groups were performed by the Tukey–Kramer test (GraphPAD Software). P values less than or equal to 0.05 were considered significant.

## Figures and Tables

**Table 1:** Oligonucleotide primer sequences used for real-time PCR.

Gene	NCBI Reference Sequence	Primer Sequence
*p53*	NM_000546.6	Forward: 5′-GTGTGGAGTATTTGGATGAC-3′ Reverse: 5′-ATGTAGTTGTAGTGGATGGT-3′
*Bax*	NM_138764.5	Forward: 5′-GGACGAACTGGACAGTAACATGG-3′ Reverse: 5′-GCAAAGTAGAAAAGGGCGACAAC-3′
*Bcl-2*	NM_000657.3	Forward: 5′-ATCGCCCTGTGGATGACTGAG-3′ Reverse: 5′-CAGCCAGGAGAAATCAAACAGAGG-3′
*Bcl-XL*	NM_138578.3	Forward: 5′-CGGTACCGGCGGGCATTCAG-3′ Reverse: 5′-CGGCTCTCGGCTGCTGCATT-3′
*CASP3*	NM_004346.4	Forward: 5′-AGCACCTGGTTATTATTCTTGG-3′ Reverse: 5′-GCTTGTCGGCATACTGTT-3′
*CASP9*	NM_001229.5	Forward: 5′-GCTCAGACCAGAGATTCG-3′ Reverse: 5′-ATCCTCCAGAACCAATGTC-3′
*β-actin*	NM_001101.5	Forward: 5′-TTGTTACAGGAAGTCCCTTGCC-3′ Reverse: 5′-ATGCTATCACCTCCCCTGTGTG-3′

## Data Availability

Not applicable.
